# Grinding Deformation Behavior of a Lamellar γ-TiAl Alloy

**DOI:** 10.3390/ma18133114

**Published:** 2025-07-01

**Authors:** Jiale Qin, Mengxi Xu, Renci Liu, Yingying Shen, Zhiqiang Shan, Zuohai Zhu, Dong Liu, Yuyou Cui, Rui Yang

**Affiliations:** 1 School of Materials Science and Engineering, University of Science and Technology of China, Shenyang 110016, China; 2 Shi-Changxu Innovation Center for Advanced Materials, Institute of Metal Research, Chinese Academy of Sciences, Shenyang 110016, Chinaryang@imr.ac.cn (R.Y.); 3 Analysis and Testing Center, Institute of Metal Research, Chinese Academy of Sciences, Shenyang 110016, China; yyshen15b@imr.ac.cn

**Keywords:** γ-TiAl alloy, grinding, surface deformation, lamellar orientation

## Abstract

γ-TiAl alloys are susceptible to surface damage during grinding, deteriorating their mechanical properties during service. However, the underlying mechanism of surface microstructure deformation during grinding remains incompletely understood. This work systematically investigated the deformation behavior of surface lamellae in a Ti-45Al-2Nb-2Mn-1B (at.%) alloy during grinding. The surface lamellae exhibit bending after grinding, with the degree of bending angle *φ* depending on the orientation of the lamellae. The bending angle *φ* depends on both the angle between the lamellae interface normal and the grinding direction, and the angle between the lamellae interface normal and the grinding surface normal. The lamellar deformation depth *h* is primarily governed by the grinding depth. The surface of the sample after grinding can be divided into three distinct layers: a surface fine-equiaxed grain zone, a bending lamella zone, and a near-surface deformation zone. The deformation in the bending lamella zone primarily results from slip bands and stacking faults, whereas the near-surface deformation zone contains extensive dislocation tangles. The results offer fundamental insights into the deformation mechanism of surface lamellar colonies during grinding and provide theoretical guidance for the machining of γ-TiAl alloy components.

## 1. Introduction

γ-TiAl alloys have attracted extensive research interest due to their outstanding properties, including low density, high specific modulus, high specific strength, and superior oxidation and corrosion resistance [1,2,3,4]. These properties, combined with their exceptional high-temperature strength retention (up to 900 °C), position γ-TiAl alloys as potential substitutes for selected superalloys in high-temperature applications (600–900 °C) [2,3,4,5]. Currently, γ-TiAl alloys are primarily used in low-pressure turbine blades for aircraft engines and automotive engine exhaust valves [6,7,8,9,10]. These engine components demand exceptionally high dimensional accuracy, requiring precision grinding processes to meet assembly standards [11,12]. However, the machinability of γ-TiAl alloys is significantly degraded by several distinctive characteristics, including high strength retention at elevated temperatures, low thermal conductivity, and a strong chemical affinity for cutting tools [13,14,15]. Furthermore, the machining of γ-TiAl alloys is more prone to severe surface and near-surface damage, characterized by microcrack initiation and propagation. Such damage degrades surface integrity, adversely affecting mechanical properties and potentially causing premature failure or rejection of components [16].

The surface machining of γ-TiAl alloys has been extensively investigated with a focus on the effects of various machining strategies on surface quality and material performance [15,16,17,18]. Klocke et al. [19] and Kolahdouz et al. [20] demonstrated that surface quality optimization can be achieved through tailored process parameters, including low-temperature machining and high-speed machining with minimal lubrication. Similarly, temperature-controlled material property modification has been applied to tool steels [21]. Zang et al. [22] and Furusawa et al. [23] independently investigated the mechanisms of surface cracking during machining-induced microstructural deformation, revealing that crack formation correlates strongly with machining-generated surface stresses. Zeppenfeld and Klocke [24] further elucidated that the formation of grinding cracks in γ-TiAl alloys originates from the distinct mechanical behavior of α_2_ and γ phases. Comprehensive studies by Hood et al. [25], Sharma et al. [13], and Klocke et al. [19] collectively contributed to a broader understanding of the machinability of γ-TiAl alloys, revealing that the depth of the deformed layer is strongly influenced by the specific machining process and method employed. Currently, the research on the surface machining of γ-TiAl alloys predominantly focuses on the surface quality control and its influence on material properties [26,27,28], whereas micro-deformation mechanisms of surface microstructures remain understudied, yet crucial. In addition, the deformation behavior of the α_2_ + γ lamellar colonies exhibits significant anisotropy [29], critically influencing surface processing quality and material properties.

This study systematically investigated the micro-deformation behavior of surface lamellae in Ti-45Al-2Nb-2Mn-1B (at.%) (45XD, hereinafter referred to as 45XD) alloy during grinding processes. Our previous work [17] demonstrated that the grinding depth has a significant impact on the surface integrity and rotational bending fatigue performance of γ-TiAl alloys. Greater grinding depths correspond to lower fatigue life, and when the grinding depth reaches 0.2 mm, the fatigue life significantly deteriorates. We have investigated the properties of TiAl alloys after grinding processing in some depth and observed the deflection of α_2_/γ lamellae after grinding [17]. Then, we considered building a new model on the orientation of the lamellar in three dimensions with respect to the direction of grinding and explored the deflection of lamellar with different orientations. Therefore, after characterizing the surface morphology of specimens subjected to varying grinding depths (shown in Supplementary Material Figure S1) and balancing machining efficiency with surface quality, we selected the 0.2 mm grinding depth specimens as the representative research objects to elucidate the micro-deformation mechanisms under surface grinding. The work established correlations between lamellar colony crystal orientation and deformation characteristics (bending angle and depth). Detailed analyses of post-grinding microscopic morphology and deformation modes were conducted, revealing mechanistic differences between surface and near-surface regions. Specific focus was placed on regional variations in deformation patterns.

## 2. Experimental Section

The 45XD alloy cast plates were fabricated through a process involving two rounds of vacuum consumable arc remelting and one round of vacuum induction skull melting, followed by the pouring of the molten metal into a ceramic mold shell with Y_2_O_3_ primary coating. The cast plates were subjected to hot isostatic pressing at 1260 °C/150 MPa for 4 h to eliminate internal porosity, and then held at 1010 °C for 8 h to relieve residual stresses. The specimens with a size of 100 × 25 × 10 mm were cut by using an electric discharge wire from the cast plates. Subsequently, the specimens were ground to a depth of 0.2 mm in a single-pass liquid-cooled grinding operation using an 80# silicon carbide grinding wheel. The grinding operation was conducted under precisely controlled parameters: the grinding wheel maintained a linear velocity of 25 m/s, and workpiece traverse speed was configured at 50 mm/min.

The near-surface region of the ground specimens was examined by an optical microscopy (OM, Axiovert 200 MAT, Zeiss, Oberkochen, Germany). Three-dimensional surface topography was reconstructed through scanning and data processing operations performed using a Micro XAM-3D (KLA Corporation, Phoenix, AZ, USA) white light Interferometer. The microhardness data of the subsurface with different orientations was measured with an LM247AT microhardness tester (LECO, St. Joseph, MI, USA). The crystallographic orientation of the surface lamellae was examined using electron backscatter diffraction (EBSD, Merlin compact, Zeiss, Oberkochen, Germany). EBSD specimens were prepared through grinding, followed by electropolishing in a solution of 10 vol.% perchloric acid, 30 vol.% n-butanol, and 60 vol.% methanol at a temperature ranging from −30 °C to −20 °C. The surface of the post-grinding specimens was observed by scanning electron microscopy (SEM, Apero, FEI, Hillsboro, OH, USA). Transmission electron microscopy (TEM, JEM-2100Plus, JEOL Ltd., Akishima City, Tokyo and FEI Talos F200X, Thermo Fisher Scientific, Waltham, MA, USA) was employed to characterize the initial microstructure of the alloy, as well as the surface microstructure after grinding. TEM specimens were thinned by ion milling. Moreover, a focused ion beam (FIB, FEI Helios NanoLab 600i, FEI, Hillsboro, OH, USA) was utilized to cut TEM specimens from the surface of the ground specimen.

## 3. Results and Discussion

### 3.1. Grinding-Induced Surface Morphology and Cross-Sectional Characteristics

**Figure 1:**
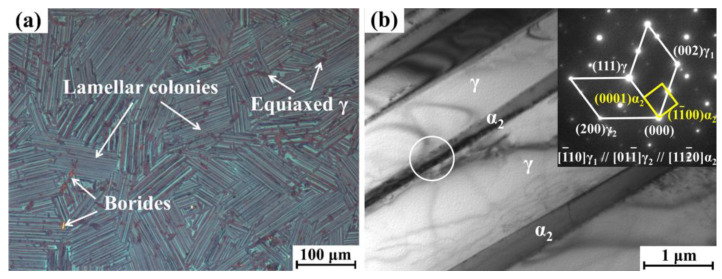
(**a**) Metallograph; (**b**) BF-TEM image exhibiting the initial microstructure of the 45XD alloy, white circles indicate the TEM spot positions in (**b**).

As illustrated in Figure 1a, the initial microstructure of the 45XD alloy specimen primarily consists of γ + α_2_ lamellar colonies with minor equiaxed γ grains and borides (TiB and TiB_2_ [30]). The γ + α_2_ lamellar colonies consist of alternating α_2_ and γ lamellae, as shown in Figure 1b.

**Figure 2:**
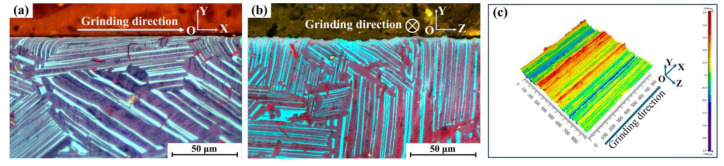
(**a**) XOY surface metallograph, (**b**) YOZ surface metallograph, and (**c**) white light interferogram exhibiting the surface morphology after grinding of the 45XD alloy.

**Figure 3:**
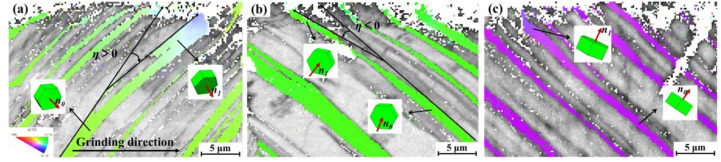
(**a**–**c**) IPF map exhibiting the cross-sectional microstructure of the 45XD alloy after grinding.

Under the influence of grinding processing with a depth of 0.2 mm, surface lamellar colonies exhibit significant deformation, as shown in Figure 2. For descriptive clarity, the grinding direction is defined as the *X*-axis, while the *Y*-axis represents the normal direction to the ground surface, and the *Z*-axis corresponds to the normality of the XOY plane. As revealed in Figure 2a, the surface lamellae undergo differential deflections with varying angular deviations following the grinding process. Specifically, as schematically illustrated in Figure 3a–c, the deflection angle *η* (defined as the angular deviation of lamellae in the XOY plane) reveals distinct directional characteristics: specific lamellae exhibit forward deflection along the grinding direction (*η* > 0), whereas others demonstrate reverse deflection relative to the grinding direction (*η* < 0). The cross-sectional microstructure along the YOZ plane, presented in Figure 2b, reveals a characteristic serrated surface profile. This morphology originates from the compressive stresses generated laterally during abrasive plowing, as previously reported in analogous grinding processes [17]. Subsequent characterization of the three-dimensional surface topography was conducted on the post-grinding specimen. The surface roughness Sa was 0.550 μm, as systematically documented in Figure 2c. A comparative analysis demonstrates that the ground surface maintains notable smoothness along the grinding direction (*X*-axis) while exhibiting pronounced periodic fluctuations perpendicular to the grinding direction (*Z*-axis). These observed topographical characteristics show precise correspondence with the cross-sectional microstructural patterns revealed in Figure 2a,b.

**Figure 4:**
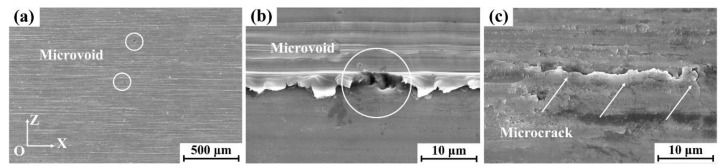
XOZ surface topography of a 45XD alloy specimen at a 0.2 mm depth of grinding: (**a**) 200× and (**b**,**c**) 10,000×.

As shown in Figure 4, the post-grinding surface morphology (XOZ plane) of the specimen was further characterized by SEM. A limited number of microvoids were identified on the processed surface, as highlighted by circular markers in Figure 4a, with Figure 4b providing a magnified view of the microvoid region. These surface microvoids likely originated from the fragmentation and spallation of boride particles during the grinding process [17]. Furthermore, microcracks were observed on the surface, as indicated by arrows in Figure 4c. The formation of these surface microcracks can be attributed to the substantial residual stresses generated during grinding operations [11,17].

### 3.2. Orientation-Dependent Evolution of Lamellar Bending Angle and Deformation Depth

The deformation behavior of α_2_ + γ lamellar colonies exhibits pronounced anisotropic characteristics [29]. The change in lamellar microstructure after grinding was judged by α_2_ phase. Plastic deformation was visible in the form of bending of the lamellae on the surface [25]. Consequently, systematic investigations were conducted to evaluate the deformation responses of lamellae with varying orientations following grinding processes. This study focuses on quantifying the angular deflection patterns and deformation depth variations in surface-layer colonies with distinct crystallographic orientations post-grinding.

**Figure 5:**
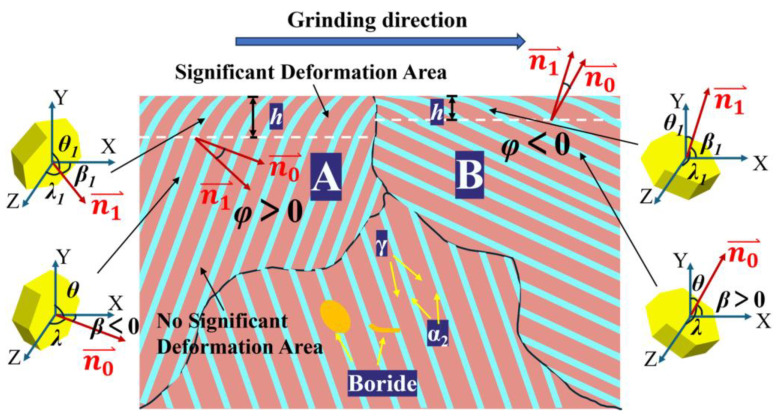
Schematic diagrams of *β*, *θ*, *λ,* schematic diagram of positive and negative *β*, deformation depth *h* schematic, and schematic diagram of positive and negative bending angle *φ*.

In order to clarify the relationship between bending angle, deformation depth, and grain orientation, a model is established as shown in Figure 5 The bending angle *φ* is defined as the angular deviation between the [0001] planes (α_2_/γ lamellar interface) of α_2_ grains before and after deformation. The lamellar deformation depth *h* represents the depth at which the lamellar begins to exhibit significant deformation, which can be measured directly. As depicted in Figure 5, for the unbent α_2_ grains, the normal vector *n*_0_ of the [0001]_α2_ plane forms angles *β*, *θ*, and *λ* with the X, Y, and Z axes, respectively. Note that *n*_0_ is the normal state of the laminar interface. After significant bending, the normal vector of the [0001]_α2_ plane of the α_2_ grain is denoted as *n*_1_, forming angles *β*_1_, *θ*_1_, and *λ*_1_ with the X, Y, and Z axes, respectively. The angle between the [0001]_α2_ planes before and after bending is referred to as the bending angle *φ*. As shown in region A of Figure 5, *φ* is positive when the lamella is deflected clockwise along the grinding direction, and negative when the lamella is deflected counterclockwise, as shown in region B. When the *n*_0_ vector is located in the positive hemisphere of the *Y*-axis, *β* is positive, as shown in Figure 3a,b. Conversely, when the *n*_0_ vector is in the negative hemisphere of the *Y*-axis, *β* is considered negative, as shown in Figure 3c.

The bending angle *φ* can be calculated as the angle between the normal vectors of the [0001]_α2_ surface before and after bending. The angles between the [0001] direction of the unbent α_2_ lamella in the surface and the three orthogonal directions were measured using the polar figure of the EBSD. The [0001] surface of α_2_-Ti_3_Al before (S_0_) and after (S_1_) the bending can be expressed as follows:

(1) $$S_{0}:\cos\beta x+\cos\theta y+\cos\lambda z=D_{0}$$

(2) $$S_{1}:\cos\beta_{1}x+\cos\theta_{1}y+\cos\lambda_{1}z=D_{1}$$

Due to the difficulty in collecting EBSD data of the α_2_-Ti_3_Al phase at the surface, caused by the influence of the grinding process, the calculation is performed using the deflection angle *η* of the lamellae in the XOY cross-section, as shown in Figure 3a. To simplify the calculation and considering the center symmetry of space, the range of the *β* angle is set to (−90°, 90°). Equation (2) is then rewritten as follows:

(3) $$S_{1}:x+B_{1}^{\prime }y+C_{1}^{\prime }z=D_{1}^{\prime }$$

By defining the angle between the unbent and bent lamellae in space as *φ*, with positive and negative values as shown in Figure 5, *φ* can be calculated using the following equation:

(4) $$\varphi =\cos^{-1}\left(\frac{\cos\beta +B_{1}^{\prime }\times \cos\theta +C_{1}^{\prime }\times \cos\lambda }{\sqrt{{cos\beta }^{2}+{cos\theta }^{2}+{cos\lambda }^{2}}\times \sqrt{1^{2}+{B_{1}^{\prime }}^{2}+{C_{1}^{\prime }}^{2}}}\right)$$

In the above equation, cos *β*, cos *θ*, and cos *λ* are known, while $B_{1}^{\prime }$ and $C_{1}^{\prime }$ are unknown. Therefore, the value of *φ* can be determined by solving for these two unknowns. $C_{1}^{\prime }$ can be calculated by considering the consistent depth of lamellae deformation. Specifically, the intersection line between the unbent and bent lamellae lies on the XOZ plane, and it is perpendicular to the vector (0,1,0). Based on this, the corresponding relationship is given by Equation (5):

(5) $$\left|\begin{array}{ccc} i & j & k\\ \cos\beta & \cos\theta & \cos\lambda \\ 1 & B_{1}^{\prime } & C_{1}^{\prime } \end{array}\right|\left(0,1,0\right)=0$$

The calculation yields the following value for $C_{1}^{\prime }$:

(6) $$C_{1}^{\prime }=\frac{\cos\gamma }{\cos\beta }$$

The process of calculating $B_{1}^{\prime }$ using the lamellae deflection angle in the XOY cross-section is outlined as follows. The projection of the normal vector *n*_0_ of the unbent plane onto the XOY cross-section is denoted as *n*_0*xy*_. Similarly, the projection of the normal vector *n*_1_ of the bent plane onto the XOY section is expressed as follows:

(7) $$\vec{n_{0xy}}=\left(\cos\beta ,\cos\theta ,0\right)$$

(8) $$\vec{n_{1xy}}=\left(1,B_{1}^{\prime },0\right)$$

The lamellae deflection angle *η* of both the unbent and bent planes within the XOY cross-section can be obtained through direct measurement of the metallographs, as shown in Figure 3a,b. Based on this angle, the value of $B_{1}^{\prime }$ can be determined as follows:

(9) $$\vec{n_{0xy}}\cdot \vec{n_{1xy}}=\left|\vec{n_{0xy}}\right|\left|\vec{n_{1xy}}\right|\cos\eta$$

The simplified arithmetic for Equation (9) is as follows:

(10) $$B_{1}^{\prime }=\frac{-b\pm \sqrt{b^{2}-4ac}}{2a}$$

(11) $$a=\left[\cos^{2}\theta -\cos^{2}\eta \left(\cos^{2}\beta +\cos^{2}\theta \right)\right]$$

(12) b=cos⁡βcos⁡θ

(13) $$c=\left[\cos^{2}\beta -\cos^{2}\eta \left(\cos^{2}\beta +\cos^{2}\theta \right)\right]$$

The $B_{1}^{\prime }$-value is further determined from the orientation relationship within the XOY cross-section. In summary, the angle *φ* between the unbent and bent lamellae layers in space can be calculated by combining Equations (4), (6), and (10). The supplementary material exemplifies this calculation process.

**Figure 6:**
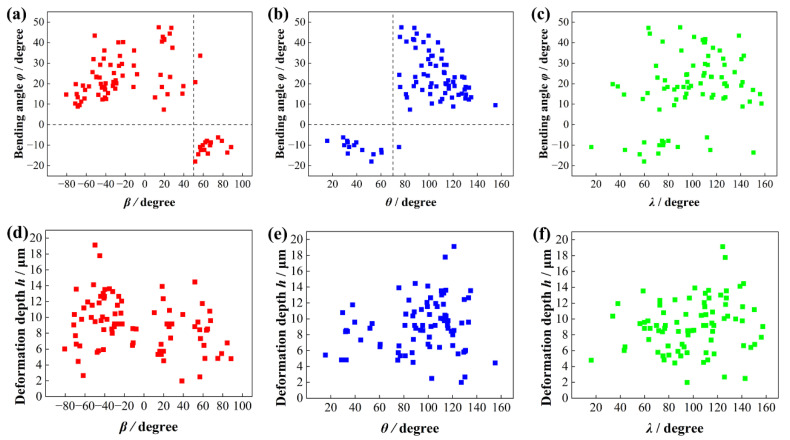
Influence of orientation angles on lamellar bending angle *φ*: (**a**) *β*, (**b**) *θ*, (**c**) *λ*, and on deformation depth *h*: (**d**) *β*, (**e**) *θ*, (**f**) *λ*.

Measurements were conducted on eight discrete samples, averaging ten measurement points per specimen. The findings are summarized as follows: as seen in Figure 6a, *φ* increases and then decreases as *β* increases from −90° to 50°. When *β* exceeds 50°, *φ* becomes negative. Figure 6b shows that *φ* initially decreases and then increases with *θ* between 0° and 70°, fluctuating around −15°. Beyond 70°, *φ* becomes positive, reaching a peak at *θ* = ~90° before decreasing. Figure 6c indicates that *φ* has little dependence on *λ*. The lamellar deformation depth exhibits minimal sensitivity to *β*, *θ*, and *λ* angles, with no significant variations observed across their ranges, as demonstrated in Figure 6d–f. During material processing operations, the specimen is subjected to a multiaxial stress state characterized by concurrent shear stress along the grinding vector and compressive stress normalized to the machined surface [31]. The *X*-axis aligns with the shear stress direction, where the *β*-angle is defined as the orientation between the normal to the [0001] plane of α_2_ and the *X*-axis. Correspondingly, the *Y*-axis coincides with the compressive stress direction, with the *θ*-angle representing the orientation of the [0001] plane normal relative to the *Y*-axis. This geometric coupling explains the dominant influence of *β* and *θ* angles on lamellar bending behavior, while *λ* is determined by these two angles. As shown in Figure 6a,b, when *β* > 50° and *θ* < 70°, the lamellar bending angle *φ* becomes negative (*φ* < 0), exhibiting reverse bending morphology exemplified in Figure 3c. This reversal behavior is predominantly influenced by compressive stress under these angular conditions. Furthermore, the lamellar deformation depth *h* is primarily governed by the grinding depth. Ni et al. [17] demonstrated a strong positive correlation between grinding depth and lamellar deformation depth *h*, the deformation depth *h* increases with grinding depth.

**Figure 7:**
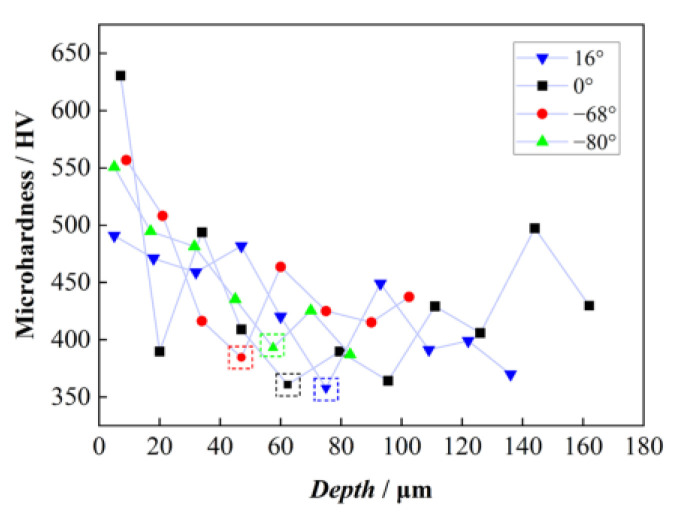
The microhardness of lamellae with different angel *β* in XOY surface (the dashed box shows the hardened layer depth points).

In order to obtain the hardness of lamellar colonies with different orientations angle *β* after grinding, the data were collected along the depth in the same oriented lamellar colony to eliminate the influence of the change in the lamellar thickness. It can be seen from Figure 7 that the maximum microhardness, above 500 HV, occurred near the surface, and then decreased with the increase in depth. The thickness of the hardened layer was between 40 μm and 80 μm. The thickness of the layer was related to the initial microstructure. The hardened layer of the lamellar colonies whose initial orientation angle *β* was 0° or 16° was slightly deeper than that of −80° and −68°. This indicated that the α_2_ + γ lamellar orientation has an effect on the depth of hardened layer on the grinding surface, the hardened layer of the lamellae perpendicular to grinding direction was deeper, while the initial lamellae parallel to the grinding direction had a thinner hardened layer.

### 3.3. Underlying Microscale Deformation Mechanisms

**Figure 8:**
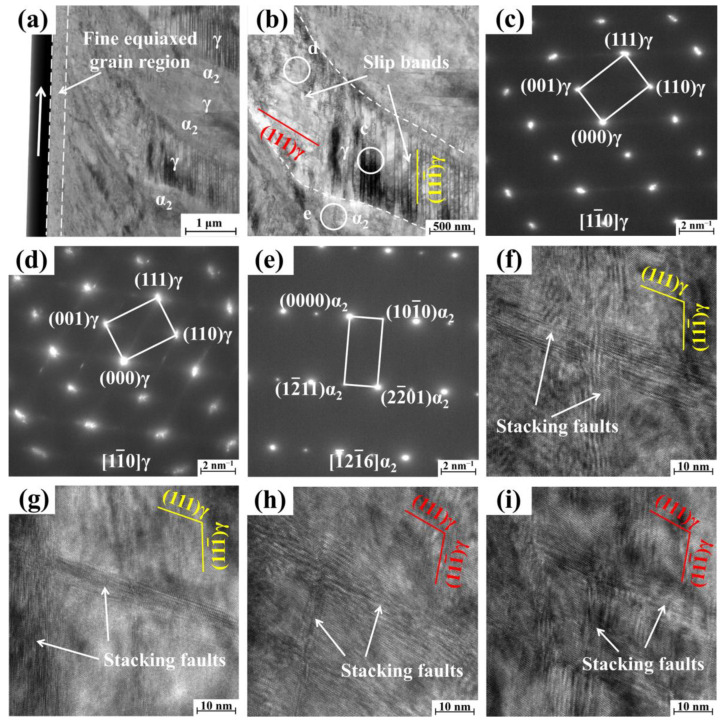
Cross-sectional microstructure after grinding. (**a**) BFSTEM image; (**b**) is a partial magnification of (**a**); (**c**–**e**) are selected area electron diffraction patterns from (**b**); and (**f**–**i**) are HR-TEM images from γ lamellae in (**b**).

To further investigate the microscopic deformation mechanisms of surface lamellar structures, TEM specimens were extracted from the ground sample surface using the focused ion beam (FIB) technique. As shown in Figure 8a, an equiaxed fine-grained region approximately 300 nm thick formed on the machined surface. Significant microstructural evolution was observed in the surface layer, indicating substantial plastic deformation and grain refinement. Previous studies have demonstrated that the peak temperature at the workpiece surface during grinding approaches 800 °C [32]. The combined effects of this elevated temperature [33] and severe surface plastic deformation triggered dynamic recrystallization in the affected areas, leading to the transformation from lamellar structures to fine equiaxed grains. Moreover, due to the low thermal conductivity of γ-TiAl alloys, the high temperature was primarily concentrated on the sample surface, while the subsurface regions maintained relatively lower temperatures [32]. Consequently, only plastic deformation without recrystallization occurred in the near-surface areas, as evidenced by the microstructural characteristics presented in Figure 8a,b. This thermal-mechanical coupling effect ultimately resulted in the formation of a relatively thin equiaxed fine-grained layer on the ground surface.

As shown in Figure 8b, bending of subsurface γ lamellae accompanied by significant crystallographic orientation changes was observed in the ground specimen. Two distinct regions of γ lamellae exhibited approximately 10.8° rotation around the [$1\bar{1}0$] crystallographic axis, as depicted in Figure 8c,d. Furthermore, numerous parallel slip bands aligned along {111}_γ_ planes were identified within the γ lamellae. The slip planes of slip bands in different regions of the γ lamellae are distinct: the slip planes in regions near the surface are along the (111)_γ_ plane, while those in regions near the interior are along the ($11\bar{1}$)_γ_ plane. The formation of stacking faults along (111)_γ_ and ($11\bar{1}$)_γ_ planes was observed within the γ lamellae, as shown in Figure 8f,i. During the grinding process, severe plastic deformation occurs on the surface and the temperature rises, which promote the formation of slip bands along {111}_γ_ planes within the γ lamellae [34,35]. In tensile, fatigue, and creep deformation of TiAl alloys, deformation primarily occurs via dislocation slip and twinning, with slip band formation rarely observed [36,37,38]. However, planar slip bands were identified on the surface of grinding material. This phenomenon is primarily attributed to the severe plastic deformation experienced during surface grinding under high strain rates. These results demonstrate that under extreme deformation conditions, TiAl alloys can deform through the formation of slip bands, deviating from conventional deformation mechanisms. Simultaneously, the intense deformation of the ground surface also promotes the formation of abundant stacking faults within γ lamellae. Numerous studies have demonstrated that the emergence of slip bands promote dislocation pile-ups and stress concentration, initiating cracks [39,40,41,42]. Therefore, in engineering components such as turbine blades, close attention should be paid to the bending lamella zone with bent γ lamellae, where slip bands may induce surface crack initiation, potentially leading to functional failure.

**Figure 9:**
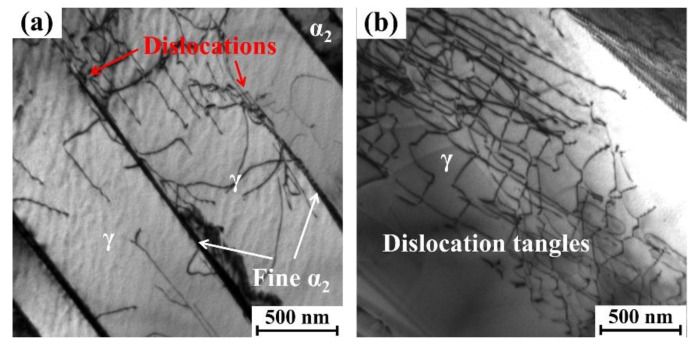
BF-TEM images exhibiting the microstructure of the near-surface after grinding, from (**a**) the surface to (**b**) the interior.

In addition, the deformation behavior in the near-surface regions without significant bending was comparatively characterized. As shown in Figure 9a, a high density of dislocations was observed within the γ lamellae. Furthermore, substantial dislocation activation was identified at the tips of α_2_ lamellae, as indicated by the red arrows in Figure 9a. In certain γ lamellae regions, extensive entanglement of dislocations with different orientations was observed, as demonstrated in Figure 9b. The deformation in these areas primarily resulted from the movement of dislocations along multiple directions, with no visible slip bands or stacking faults observed. This phenomenon is likely attributed to the relatively low deformation degree in these regions.

**Figure 10:**
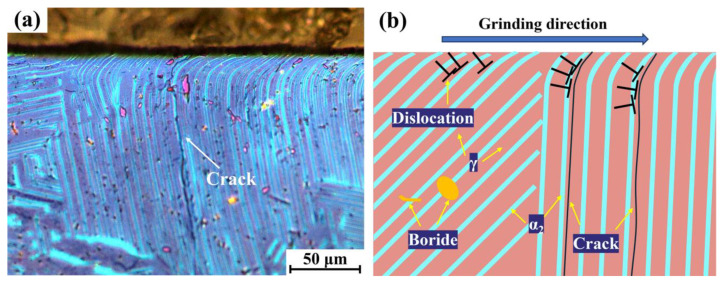
(**a**) Metallographic picture of cracks on the surface after grinding and (**b**) schematic diagram of crack initiation and extension.

As evidenced in Figure 10a, macrocracks were observed in surface lamellae perpendicular to the grinding direction (*β* = 0°). These cracks initiated at the ground surface and propagated on the subsurface along α_2_/γ interfaces. In the bending lamella zone, severe deformation occurred, leading to the formation of slip bands within the γ lamellae, as shown in Figure 8b. Due to the deformation mismatch between the α_2_ and γ phases, these slip bands readily cause dislocation pileups at the γ/α_2_ interfaces, subsequently triggering crack initiation, as shown in Figure 10b. Consequently, the presence of bent lamellar regions constitutes the primary cause of performance degradation in grinding TiAl alloy components, and minimizing the occurrence of such regions is essential. As demonstrated in the study by Ni et al. [17], the rotating bending fatigue life of TiAl samples significantly decreased after surface grinding. Furthermore, our findings indicate that lamellae oriented nearly perpendicular to the grinding direction (*β* = 0°) exhibit the maximum bending angles. This orientation experiences more severe deformation and is more prone to cracking; thus, situations where the grinding direction is perpendicular to the lamellae should be minimized. Specifically, for TiAl components with specific orientations, such as single-crystal TiAl components [43] and suction casting TiAl components [44], the grinding direction should be controlled to be parallel to the lamellar planes during surface grinding. This minimizes the bending degree of surface lamellae, thereby preserving the alloy’s performance.

## 4. Conclusions

This work investigated the surface microstructural deformation of 45XD alloy under grinding. The main findings are as follows:

1. The model is established to describe the lamellar bending angle. *φ* is governed by the coupling between interface normal-grinding direction angle *β* and interface normal-surface normal angle *θ*, with *β* > 50 triggering reverse bending.
2. The ground surface exhibits distinct stratified deformation. Fine equiaxed grains form in conditions of high temperature and strain on the surface. The bending lamella zone is dominated by {111}_γ_ slip bands and stacking faults. The near-surface zone with dislocation tangles and α_2_-tip activation, devoid of recrystallization.
3. Critical crack-orientation relationship: Severe bending at *β* = 0 (lamellae perpendicular to grinding) triggers dislocation pileups at α_2_/γ interfaces, exclusively initiating cracks that propagate subsurface. This defines a high-risk orientation for component failure.

## Data Availability

The original contributions presented in this study are included in the article. Further inquiries can be directed to the corresponding author.

## Supplementary Materials

The following supporting information can be downloaded at https://www.mdpi.com/article/10.3390/ma18133114/s1, Figure S1: Surface topography of 45XD alloy samples with different grinding depths: (a,b) 0.1 mm, (c,d) 0.2 mm, (e,f) 0.5 mm, (g,h) 1 mm; Figure S2: IPF and Pole figure showing the microstructure of the 45XD alloy cross-section after grinding.

## References

[1] Dimiduk D.M. (1999). Gamma titanium aluminide alloys-an assessment within the competition of aerospace structural materials. Mater. Sci. Eng. A.

[2] Kim Y.W. (1989). Intermetallic alloys based on gamma titanium aluminide. JOM.

[3] Gao G.F., Fu Z.X., Wang Y., Xiang D.H., Zhao B. (2021). Research progress on precision machining of Ti-Al intermetallic compounds. Rare Met. Mater. Eng..

[4] Kumpfert J. (2001). Intermetallic alloys based on orthorhombic titanium aluminide. Adv. Eng. Mater..

[5] Appel F., Wagner R. (1998). Microstructure and deformation of two-phase γ-titanium aluminides. Mat. Sci. Eng. R.

[6] Genc O., Unal R. (2022). Development of gamma titanium aluminide (γ-TiAl) alloys: A review. J. Alloys Compd..

[7] Loria E.A. (2000). Gamma titanium aluminides as prospective structural materials. Intermetallics.

[8] Bewlay B.P., Nag S., Suzuki A., Weimer M.J. (2016). TiAl alloys in commercial aircraft engines. Mater. High Temp..

[9] Kim Y.W. (1994). Ordered Intermetallic Alloys, Part III: Gamma titanium aluminides. JOM.

[10] Yang R. (2015). Advances and challenges of TiAl base alloys. Acta Metall. Sin..

[11] Chen T., Wang X.W., Zhao B., Ding W.F., Xu J.H. (2024). Surface integrity evolution during creep feed profile grinding of γ-TiAl blade tenon. Chin. J. Aeronaut..

[12] Hood R., Lechner F., Aspinwall D.K., Voice W. (2007). Creep feed grinding of gamma titanium aluminide and burn resistant titanium alloys using SiC abrasive. Int. J. Mach. Tool. Manuf..

[13] Sharman A.R.C., Aspinwall D.K., Dewes R.C., Bowen P. (2001). Workpiece surface integrity considerations when finish turning gamma titanium aluminide. Wear.

[14] Aust E., Niemann H.R. (1999). Machining of γ-TiAl. Adv. Eng. Mater..

[15] Ezugwu E.O., Wang Z.M. (1997). Titanium alloys and their machinability-a review. J. Mater. Process. Technol..

[16] Xia Z.W., Shan C.W., Zhang M.H., Cui M.C., Luo M. (2023). Machinability of γ-TiAl: A review. Chin. J. Aeronaut..

[17] Ni M.J., Liu R.C., Zhou H.H., Yang C., Ge S.Y., Liu D., Shi F.L., Cui Y.Y., Yang R. (2024). Influence of grinding depth on the surface integrity and fatigue property of γ-TiAl alloy. Acta Metall. Sin..

[18] Bentley S.A., Goh N.P., Aspinwall D.K. (2001). Reciprocating surface grinding of a gamma titanium aluminide intermetallic alloy. J. Mater. Process. Technol..

[19] Klocke F., Settineri L., Lung D., Priarone P.C., Arft M. (2013). High performance cutting of gamma titanium aluminides: Influence of lubricoolant strategy on tool wear and surface integrity. Wear.

[20] Kolahdouz S., Hadi M., Arezoo B., Zamani S. (2015). Investigation of surface integrity in high speed milling of gamma titanium aluminide under dry and minimum quantity lubricant conditions. Proc. CIRP..

[21] Krbata M., Kohutiar M., Escherova J., Klučiar P., Studeny Z., Trembach B., Beronská N., Breznická A., Timárová Ľ. (2025). Continuous Cooling Transformation of Tool Steels X153CrMoV12 and 100MnCrW4: Analysis of Microstructure and Hardness Changes. Appl. Mech..

[22] Zang H., Wise M.L.H., Aspinwall D.K. (1995). The surface quality of hipped gamma titanium aluminide bar after turning. Proceedings of the Thirty-First International Matador Conference.

[23] Furusawa T., Hino H., Tsuji S., Koroyasu S., Ichikawa A. (2004). Generation of defects due to machining of TiAl intermetallic compound and their effects on mechanical strength. ASME. J. Manuf. Sci. Eng..

[24] Zeppenfeld C., Klocke F. (2006). Speed stroke grinding of γ-titanium aluminides. CIRP Ann..

[25] Hood R., Aspinwall D.K., Sage C., Voice W. (2013). High speed ball nose end milling of γ-TiAl alloys. Intermetallics.

[26] Wang Z.H., Liu Y.W. (2020). Study of surface integrity of milled gamma titanium aluminide. J. Manuf. Process..

[27] Mantle A.L., Aspinwall D.K. (2001). Surface integrity of a high speed milled gamma titanium aluminide. J. Mater. Process. Technol..

[28] Pérez R.G.V. (2005). Wear mechanisms of WC inserts in face milling of gamma titanium aluminides. Wear.

[29] Paidar V., Inui H., Kishida K., Yamaguchi M. (1997). Dislocation dissociation in TiAl alloys. Mater. Sci. Eng. A.

[30] Cao R.X., Liu R.C., Yang C., Cui Y.Y., Yang R. (2023). Structures and formation mechanism of borides with varied morphologies in cast γ-TiAl alloys. Mater. Des..

[31] Wang D., Chen L., Zhang Z.P. (2024). Study on Force Model and Surface Integrity of Cylindrical Grinding 18CrNiMo7-6 Steels. China Mech. Eng..

[32] Xi X.X., Chen T., Ding W.F. (2020). Research on temperature field during grinding of low-pressure turbine blade tenon of TiAl alloys. Diam. Abras. Eng..

[33] Tian S.W., He A.R., Liu J.H., Zhang Y.F., Zhang S.Y., Zhang Y., Yang Y.G., Jiang H.T. (2021). Investigation on the microstructure evolution and dynamic recrystallization mechanisms of TiAl alloy at elevated temperature. J. Mater. Res. Technol..

[34] Mazánová V., Heczko M., Miao J.S., Mills M.J., Murphy-Leonard A. (2024). The role of coherent nano precipitates on stacking fault and deformation twin formation during tensile deformation of wire arc additive manufactured nickel-aluminum-bronze. Mater. Sci. Eng. A.

[35] Jiang Z., Lei C.Q., Ding J.J., Zhu C.N., Shi D.F., Zhang J., Wang G.Q. (2024). Synergistic effect of orientation and temperature on slip behavior and precipitation behavior of Al-Cu-Li single crystals. Trans. Nonferr. Met. Soc..

[36] Yue H.Y., Peng H., Su Y.J., Wang X.P., Chen Y.Y. (2021). Microstructure and high-temperature tensile property of TiAl alloy produced by selective electron beam melting. Rare Met..

[37] Malaplate J., Caillard D., Couret A. (2005). Correlation between creep activation parameters and microscopic dislocation behaviour in γ TiAl alloys. Mater. Sci. Eng. A.

[38] Xu X.S., Ding H.S., Huang H.T., Liang H., Ramanujan R.V., Chen R.R., Guo J.J., Fu H.Z. (2023). Twinning-induced dislocation and coordinated deformation behavior of a high-Nb TiAl alloy during high-cycle fatigue. Int. J. Fatigue..

[39] Guo H., Zhang M.M., Xu D.S., Zhang J.H., Qiu J.K., Meng Z.C., Zheng S.J., Ma Y.J., Wang H., Yang R. (2023). Planar slip triggered by successive dislocation-precipitate interaction in titanium alloys. Mater. Sci. Eng. A.

[40] Worsnop F.F., Lim R.E., Bernier J.V., Pagan D.C., Xu Y.L., McAuliffe T.P., Rugg D., Dye D. (2022). The influence of alloying on slip intermittency and the implications for dwell fatigue in titanium. Nat. Commun..

[41] Yu S.W., An X.L., Ni S., Song M. (2021). Effects of strain rate and strain on microstructural evolution and mechanical properties of a Ti-10 at.%Al alloy. Mater. Charact..

[42] Youssef S.S., Zheng X.D., Qi M., Ma Y.J., Huang S.S., Qiu J.K., Zheng S.J., Lei J.F., Yang R. (2021). Effects of Al content and α_2_ precipitation on the fatigue crack growth behaviors of binary Ti-Al alloys. Mater. Sci. Eng. A.

[43] Zhao Z.Q., Chu L.L., Yu M.L., Guo W.L., Zhang Z.H. (2024). Advanced TiAl Based Alloys: From Polycrystals to Polysynthetic Twinned Single Crystals. Adv. Funct. Mater..

[44] Xia Z.Z., Liu R.H., Shen Y.Y., Mohammed A., Jia Q., Cui Y.Y., Yang R. (2022). Creep properties of Ti–48Al–2Cr–2Nb alloy having similarly oriented lamellae with fine lamellar spacing. Mater. Sci. Eng. A.

