# Supplementary material for: Grinding Deformation Behavior of a Lamellar γ-TiAl Alloy
Source: Materials (Basel). 2025 Jul 1;18(13):3114. doi: 10.3390/ma18133114 (PMC12251003; doi:10.3390/ma18133114)
Supplement: Supplementary file 1 [file materials-18-03114-s001.zip › materials-3668994-supplementary.pdf]

Supplementary Material

Grinding deformation behavior of a lamellar γ-TiAl alloy

Jiale Qin [a,b], Mengxi Xu [a,b], Renci Liu [b,*], Yingying Shen [c], Zhiqiang Shan [b], Zuohai Zhu [b], Dong Liu [b], Yuyou Cui [b], Rui Yang [b]

[a] School of Materials Science and Engineering, University of Science and Technology of China, Shenyang 110016, China

[b] Shi-Changxu Innovation Center for Advanced Materials, Institute of Metal Research, Chinese Academy of Sciences, Shenyang 110016, China

[c] Analysis and Testing Center, Institute of Metal Research, Chinese Academy of Sciences, Shenyang 110016, China

* Corresponding author.

E-mail address: rcliu@imr.ac.cn (R.C. Liu).

As illustrated in Figure S1, at grinding depths of 0.2 mm or less, the sample surface remains relatively smooth and flat, with only some microcracks present. Increasing the grinding depth to 0.5 mm induces the formation of pronounced cracks on the surface. Furthermore, at a grinding depth of 1 mm, crack propagation is observed with concomitant material spallation.

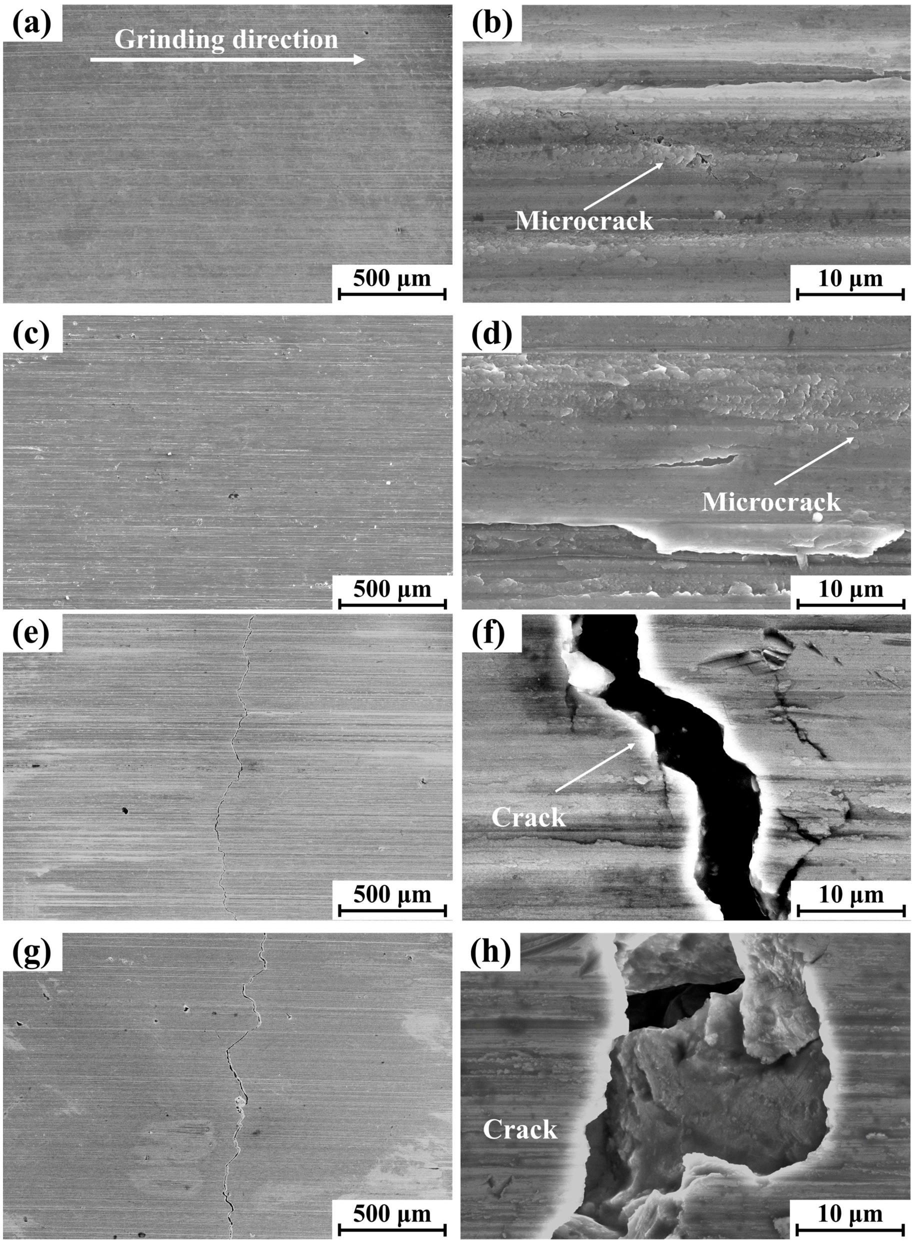

Figure S1 Surface topography of 45XD alloy samples with different grinding depths: (a, b) 0.1 mm, (c, d) 0.2mm, (e, f) 0.5mm, (g, h) 1mm.

As shown in Figure S2, the orientation angles β = -64.57°, θ = 115.45°, and λ = 42.9° of the α_2 grains in the lamella before significant deformation can be measured by pole figure, and η = 16.3° can be measured directly from Figure S2. In the formula, cos β = 0.42941, cos θ = -0.42972, cos λ = 0.73254, cos η = 0.95981.

The [0001] surface of α_2-Ti_3Al before (S_0) and after (S_1) the bending can be expressed as follows:

$$S_0: 0.42941x - 0.42972y + 0.73254z = D_0 \tag{1}$$

$$S_1: \cos\beta_1\, x + \cos\theta_1\, y + \cos\lambda_1\, z = D_1 \tag{2}$$

Equation (2) is then rewritten as follows:

$$S_1: x + B_1'y + C_1'z = D_1' \tag{3}$$

φ can be calculated using the equation (4):

$$\varphi = \cos^{-1}\left(\frac{0.42941 + B_1' \times (-0.42972) + C_1' \times 0.73254}{\sqrt{0.42941^2 + (-0.42972)^2 + 0.73254^2} \times \sqrt{1^2 + {B_1'}^2 + {C_1'}^2}}\right) \tag{4}$$

If we get the unknowns B_1' and C_1' we can calculate angle φ.

C_1' can be calculated by considering the consistent depth of lamellae deformation. Specifically, the intersection line between the unbent and bent lamellae lies on the XOZ plane, and it is perpendicular to the vector (0,1,0).

$$\begin{vmatrix} i & j & k \\ 0.42941 & -0.42972 & 0.73254 \\ 1 & B_1' & C_1' \end{vmatrix} (0,1,0) = 0 \tag{5}$$

$$C_1' = \frac{\cos\gamma}{\cos\beta} = 1.7059 \tag{6}$$

The projection of the normal vector n_0 of the unbent plane onto the XOY cross-section is denoted as n_{0xy} and the angle between the projection of the normal vector n_1 of the bent plane onto the XOY cross-section is η:

$$\overrightarrow{n_{0xy}} = (0.42941, -0.42972, 0) \tag{7}$$

$$\overrightarrow{n_{1xy}} = (1, B_1', 0) \tag{8}$$

$$\overrightarrow{n_{0xy}} \cdot \overrightarrow{n_{1xy}} = \left|\overrightarrow{n_{0xy}}\right|\left|\overrightarrow{n_{1xy}}\right| * 0.95981 \tag{9}$$

Considering the direction of lamellar deflection and connecting Equations. (7-9) can solve for B_1' = -1.8281.

C_1' = 1.7059, B_1' = -1.8281, cos β = 0.42941, cos θ = -0.42972, cos λ = 0.73254.

Then we can get φ = 15.91°.

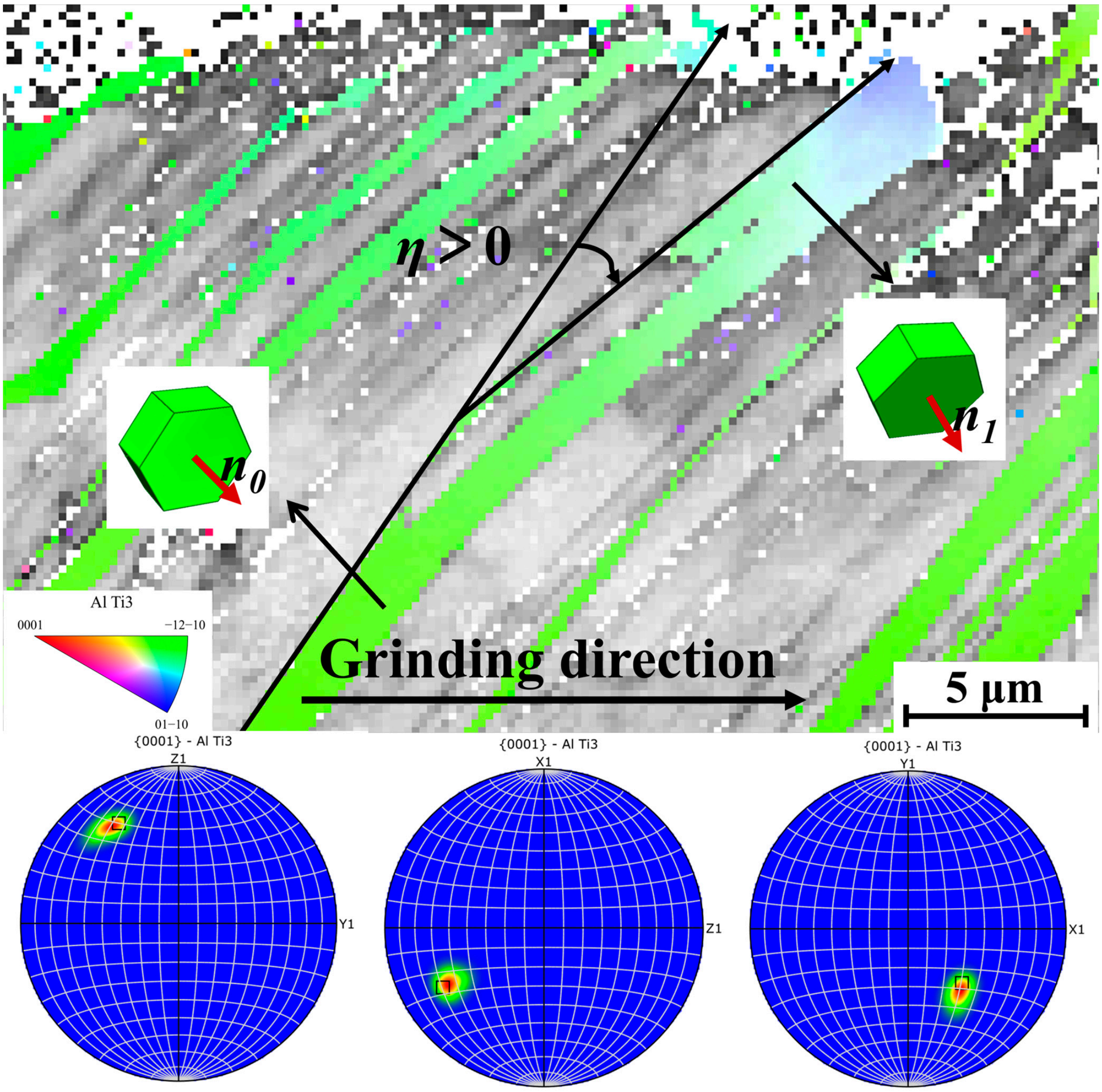

Figure S2 IPF and Pole figure showing the microstructure of the 45XD alloy cross-section after grinding.
